# Correlation between multifocal electroretinogram and optical coherence tomography findings with visual acuity after vitrectomy surgery for retinal detachment: an observational study

**DOI:** 10.1186/s40942-024-00527-7

**Published:** 2024-01-23

**Authors:** Asmaa Hassan, Mahmoud Abdel-Radi, Mohamed Omar M Aly, Sara Alattar

**Affiliations:** 1 Department of Ophthalmology, New Valley University, New Valley, Egypt; 2 Department of Ophthalmology, Assiut University, Assiut, Egypt

**Keywords:** Retinal detachment, mf-ERG, OCT, CDVA, Vitrectomy

## Abstract

**Background:**

Despite the marked increase in the anatomical success rates of macula-off rhegmatogenous retinal detachment (RRD) surgery, patients may still complain about unsatisfactory visual outcome. This study aims to correlate the postoperative corrected distance visual acuity (CDVA) with the mf-ERG (multifocal electroretinogram) and OCT (optical coherence tomography) findings following vitrectomy surgery for RRD.

**Patients and methods:**

This retrospective observational study included 40 eyes of 40 patients who underwent successful vitrectomy surgery for macula-off RRD. CDVA, mf-ERG amplitudes, mf-ERG latencies, the central macular thickness (CMT) and the integrity of the inner segment/outer segment (IS/OS) junction assessed by OCT, were evaluated 6 months postoperatively. The correlations between CDVA with mf-ERG amplitudes, mf-ERG latencies, central macular thickness, and IS/OS junction integrity were analyzed.

**Results:**

There was a statistically significant moderate positive correlation between CDVA of the studied eyes with mf-ERG amplitudes of N1, P1 and N2 in ring 1 (*P* = 0.008; *P* < 0.001 and *P* = 0.004, respectively), CMT (*P* < 0.001), and the integrity of IS/OS junction (*P* < 0.001). There was no significant correlation between CDVA and mf-ERG latencies in ring 1 (*P* > 0.05). Linear regression analysis revealed that CDVA was significantly associated with mf-ERG amplitudes and the IS/OS junction integrity. In addition, there was a strong positive correlation between mf-ERG amplitudes in ring 1 and the IS/OS junction integrity.

**Conclusions:**

The integrated interpretation of postoperative CDVA, multifocal ERG parameters, and OCT findings provides useful information about functional visual recovery and retinal microstructural changes following vitrectomy for macula-off RRD surgery. The positive correlation between the IS/OS junction integrity and the mf-ERG amplitudes was stronger than the correlation between the IS/OS junction integrity and CDVA suggesting that mf-ERG may be superior to CDVA in reflecting the extent of microstructural damage in the photoreceptor layer.

**Trial Registration:**

Clinicaltrials.gov, NCT05993208. Registered 15 August 2023 - Retrospectively registered, https://classic.clinicaltrials.gov/ct2/show/NCT05993208.

## Background

Retinal detachment (RD) represents the separation of the neurosensory retina from the underlying retinal pigment epithelium with resultant outer retinal ischemia, degeneration and loss of the photoreceptor outer segments [1]. The detachment duration and macular involvement are the most important factors that affect the visual prognosis following RD surgery [2]. In spite of the high rates of successful retinal reattachment after pars plana vitrectomy (PPV), the functional and visual recovery may be incomplete [2, 3]. Poor visual recovery may be attributed to microstructural macular changes that might be difficult to detect using standard clinical examination [4].

Several studies investigated the utilization of high-resolution spectral-domain optical coherence tomography (SD-OCT) following RD surgery to evaluate the OCT findings following RD repair and their correlation with the postoperative visual outcomes [4, 5]. Unsatisfactory visual outcomes were found to be associated with abnormal OCT findings in the form of loss of the external limiting membrane (ELM) and ellipsoid zone (EZ) integrity, cystoid macular edema (CME), epiretinal membrane, and sub-retinal fluid [4]. Similarly, other studies investigated the use of multifocal electroretinogram (mf-ERG) as a useful non-invasive tool to objectively assess the retinal function in post-vitrectomy cases [6, 7].

There are few studies regarding the use of either OCT or mf-ERG for correlation between macular microstructure and function after vitrectomy for RD. This study aims to correlate both the mf-ERG and OCT findings with the visual acuity following vitrectomy surgery for macula-off rhegmatogenous retinal detachment (RRD).

## Patients and methods

### Study design and settings

This was a retrospective observational study conducted at Assiut University Hospital and Tiba Eye Center (private practice), Assiut, Egypt.

### Ethical approval

Ethical approval was obtained from the Ethical Committee of the Faculty of Medicine, Assiut University, Egypt (IRB local approval number: 04-2023-300208). The study adhered to the tenets of the Declaration of Helsinki and was registered at Clinicaltrials.gov: NCT05993208. A written informed consent to participate in the study was obtained from all patients or their parents if the patient was below 18 years of age.

### Patient selection and assessment

The study included all patients older than 12 years who presented 6 months after vitrectomy surgery for macula-off rhegmatogenous retinal detachment for follow up. The medical records of the patients were evaluated retrospectively. Patients with incomplete medical records or lack of reliable clinical data and those who had complicated or recurrent vitrectomy surgery, scleral buckling, tractional RD, significant diabetic retinopathy or maculopathy, media opacities or other ocular diseases were excluded from this study. History was taken to evaluate the etiology of vitrectomy, history of diabetes, and duration of surgery. Clinical assessment of enrolled patients included slit lamp biomicroscopy (Haag-Streit GAT, Köniz, Switzerland) and intraocular pressure (IOP) measurement (Goldman Applanation tonometer, Haag-Streit GAT, Köniz, Switzerland). CDVA was measured for all patients utilizing Snellen’s acuity chart converted to decimal notation.

### Multifocal electroretinogram (mf-ERG)

**Fig. 1:**
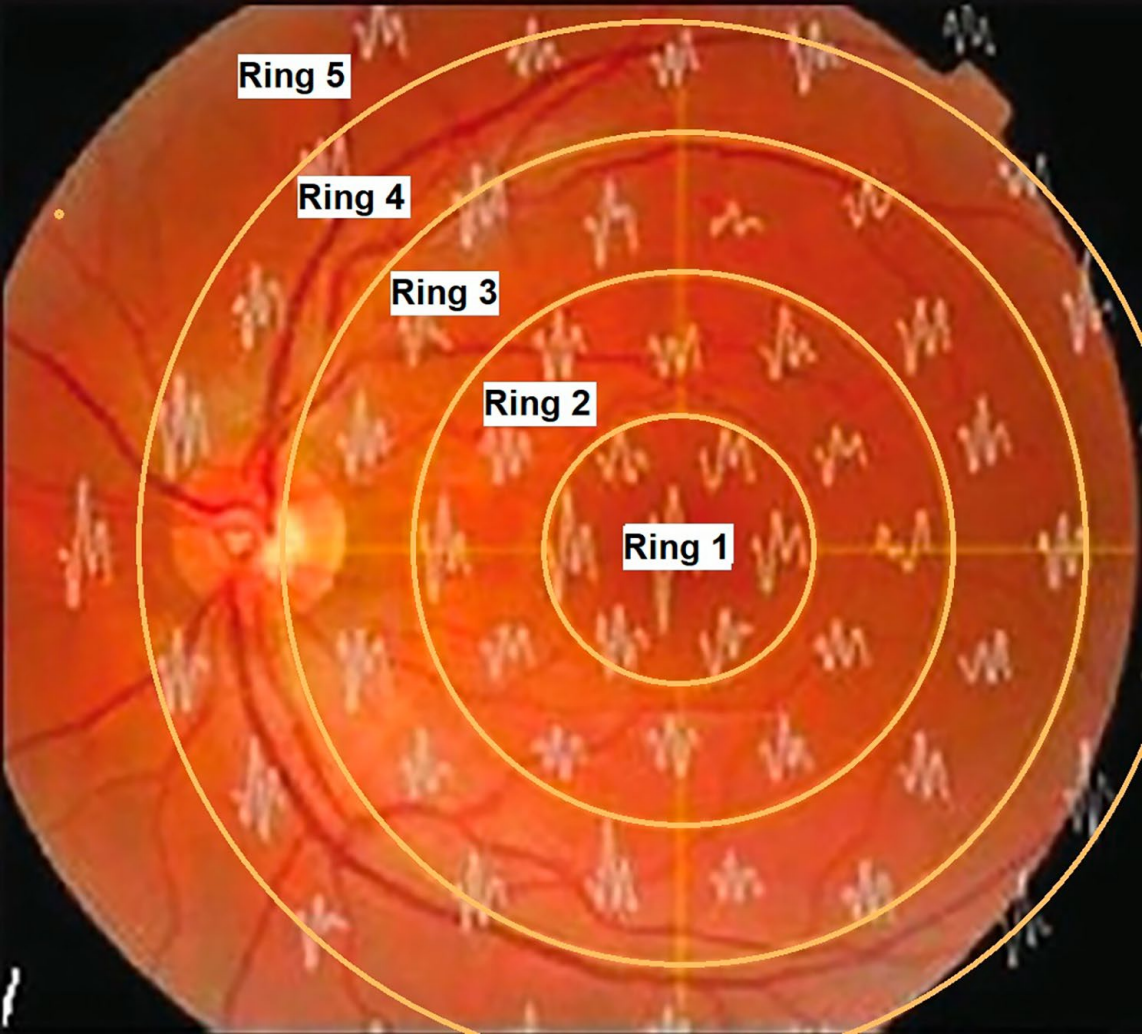
A demonstrative diagram showing the anatomic location of the 5 rings of mf-ERG stimuli projected onto the fundus. (mf-ERG: multifocal Electroretinogram)

The investigatory assessment included (mf-ERG) of both eyes that was performed using the Metrovision system (Vision Monitor, Perenchies, France) with scaled hexagons stimulating 61 zones. International Society for Clinical Electrophysiology of Vision (ISCEV) guidelines for recording the mf-ERG were followed [8]. Disposable monopolar scleral lenses (ERG jet electrode) and skin electrodes were used. At the default viewing distance, the stimulated field was ± 30° horizontally and ± 24° vertically centered on the fovea. The central hexagon subtended an angle of 3.4° at a viewing distance of 30 cm, with increasing areas subtended by peripheral hexagons. The luminance of stimulation was 100 cd/m2, the stimulus screen being surrounded by a uniformly illuminated background cover with the luminance set at 30 cd/m2 to eliminate the rod responses. Stimuli were provided by a television monitor placed 30 cm before the test eye. The stimulus frequency was set at 17 Hz. Video monitoring based on a near-infra-red sensor that records the image of the eye was used for monitoring fixation based on the Hirschberg principle. Five thousand responses were acquired for 5 min for each eye. The first-order kernel mf-ERG responses were measured. The measured waveform components in mf-ERG include the N1-wave which is the initial negative deflection corresponding to cone photoreceptor cell activity (hyperpolarization of cone photoreceptors) that largely measures the outer retinal function. The P1-wave is the positive deflection following the N1-wave representing the depolarization of inner retinal Müller cells, bipolar cells and amacrine cells providing a measure of phototransduction activity. The amplitude (Maximal light-induced electrical response generated by the various retinal cells, amplitude per unit retinal area, nV/deg^2^) and the implicit times/latency (Time needed for the electrical response to reach maximum amplitude, milliseconds) of the first negative wave for ring 1 (N1 and N2 respectively), the first positive wave for ring 1 (P1), were recorded. The amplitudes and implicit times were grouped into five rings (Fig. 1, Metrovision system): The mf-ERG stimuli corresponded roughly as follows: ring 1 to the fovea (0°–2°), ring 2 to the parafovea (2°–7°), ring 3 to the perifovea (7°–13°), ring 4 to the near periphery (3°–22°), and ring 5 to the central part of the middle periphery (22°–30.5°). Data analysis and correlations in this study were restricted to ring 1 values (central 2°). One investigator analyzed the mf-ERG responses for all patients.

### Optical coherence tomography (OCT)

All participants were subjected to OCT (Heidelberg, GmbH, Wetzlar, Germany) examination. After foveolar fixation was achieved, the device generates 40,000 scans/sec. with an axial resolution of 7 μm and a transverse resolution of 14 μm. Three scans were captured and only scans with good quality were analyzed. The central macular thickness was measured utilizing the macular thickness map protocol. The inner segment/outer segment junction was assessed by three investigators to reach an agreement of its integrity. A 6-mm horizontal line scan through the fovea was captured and 3 distinct lines with back reflection corresponding to the external limiting membrane (ELM), the IS/OS junction and the retinal pigment epithelium/Bruch’s membrane, were assessed. The integrity of the IS/OS junction, recognized as the back-reflection line corresponding to the photoreceptor IS/OS junction, was graded on 3-step scale as (normal when completely continuous / moderate disruption when partly disrupted / total loss when completely disrupted) [9]. To be expressed as a numerical variable, normal IS/OS junction was given a score of 1, moderate disruption and total loss were given scores of 0.5 and zero, respectively.

### Statistical analysis

Data were analyzed using the Statistical Package for Social Science (SPSS), version 26.0 for Windows. Qualitative data were expressed as frequency and percentages. Quantitative data were tested for normality by the Shapiro-Wilk test and expressed as mean ± SD or SE (Standard deviation / Standard of error). Independent Sample t-test was used to compare the mean difference between groups, and the Chi-Square test was used to compare proportions between groups. Spearman’s correlation was used to explore the correlations between outcome variable CDVA and exploratory variables mf-ERG, CMT, and the IS/OS junction integrity. *P* < 0.05 was considered statistically significant.

## Results

**Fig. 2:**
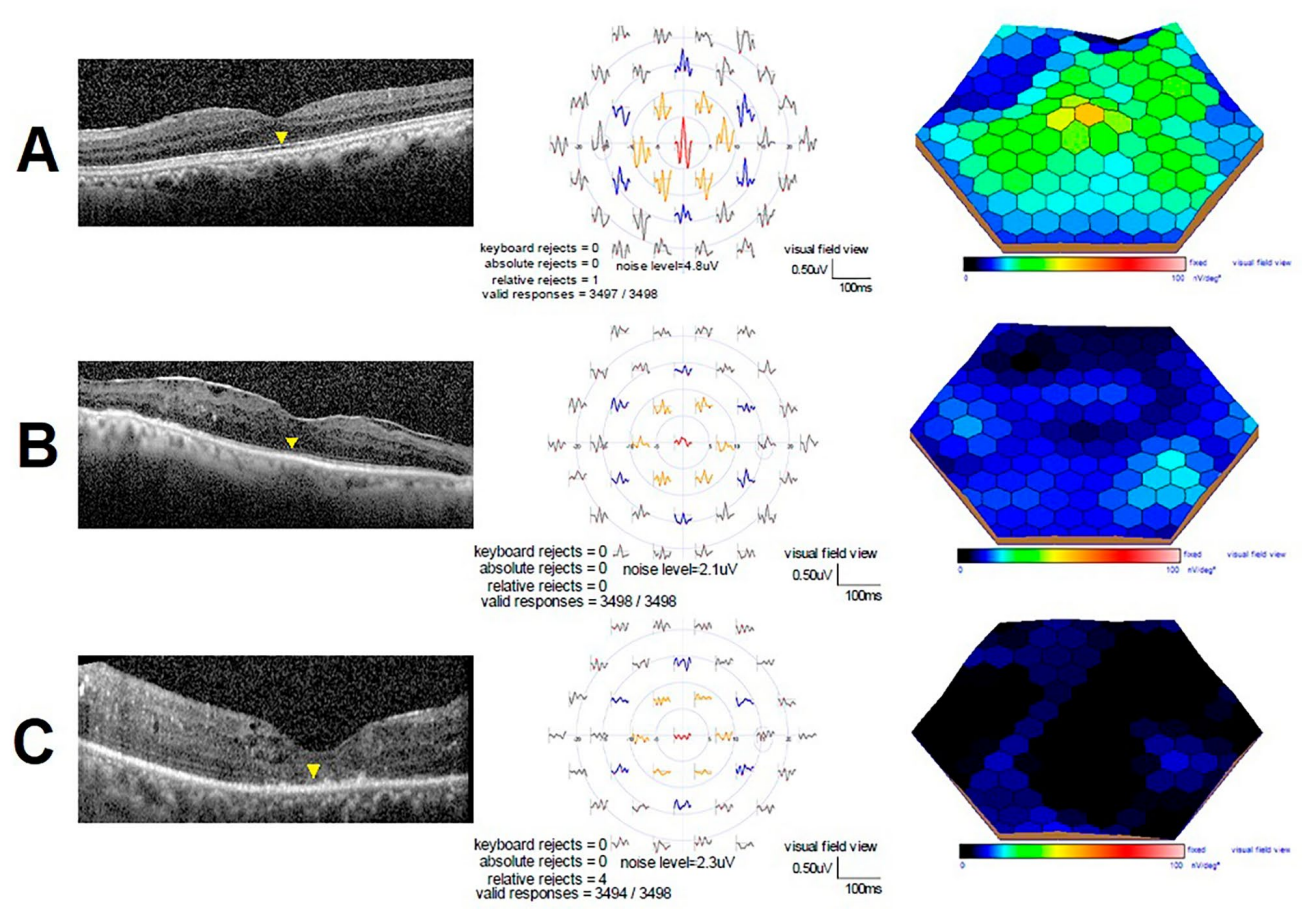
Grades of IS/OS junction integrity and their corresponding mf-ERG, 6 months postoperatively. **A**: normal IS/OS junction (OCT image-left) (yellow arrow head), trace array (middle) and 3-D topography (right) obtained from a 25-year-old male patient; **B**: moderately disrupted IS/OS junction (OCT image-left) (yellow arrow head), trace array (middle) and 3-D topography (right) obtained from a 49-year-old male patient; **C**: completely disrupted IS/OS junction (OCT image-left) (yellow arrow head), trace array (middle) and 3-D topography (right) obtained from a 45-year-old female patient. (OCT: optical coherence tomography; IS/OS: Inner segment/outer segment)

The study included 40 eyes of 40 patients (26 males and 14 females) who completed 6 months follow-up after vitrectomy surgery for macula-off rhegmatogenous retinal detachment. The mean age of participants was 48 ± 14.03 years. Sixteen patients (40%) had type 2 diabetes mellitus. The mean CDVA was 0.15 ± 0.02 (decimal notation) and the mean mf-ERG amplitude of N1 in ring 1 was 786.58 ± 94.18 nV/deg^2^. Fourteen patients (35%) had totally disrupted IS/OS junction, 17 patients (42.5%) had moderately disrupted IS/OS junction and 9 patients (22.5%) had normal IS/OS junction (Fig. 2). The mean CDVA, mf-ERG amplitude & latency values, CMT and the grading of the IS/OS junction integrity of the studied eyes, 6 months postoperatively, are summarized in Table 1.

**Table 1:** The distribution of 6-month postoperative CDVA, mf-ERG amplitude & latency values, CMT and the IS/OS junction integrity of the studied eyes

Parameters	Mean ± SD (range)
▪ CDVA (decimal notation) **†**	0.15 ± 0.02 (0.05–0.50)
▪ mf-ERG Amplitude of N1 in ring 1(nV/deg^2^) **†**	786.58 ± 94.18 (33-2680)
▪ mf-ERG Amplitude of P1 in ring 1(nV/deg^2^) **†**	1284.38 ± 870.63 (183–3000)
▪ mf-ERG Amplitude of N2 in ring 1(nV/deg^2^) **†**	1193.88 ± 155.0 (47-3592)
▪ mf-ERG Latency of N1 in ring 1(ms) **†**	27.63 ± 4.15 (14–40)
▪ mf-ERG Latency of P2 in ring 1(ms) **†**	45.98 ± 7.35 (32–65)
▪ mf-ERG Latency of N2 in ring 1(ms) **†**	65.38 ± 12.09 (40–91)
▪ CMT (um) **†**	187.20 ± 92.89 (90–637)
▪ IS/OS junction integrity	
▪ Total loss	14 (35.0%)
▪ Moderate loss	17 (42.5%)
▪ Normal	9 (22.5%)
▪ IOP**†**	15.18 ± 3.85 (11–27)

**†** Data were expressed as mean ± SD and (range) or frequency and (percentage %). CDVA: Corrected distance visual acuity; mf-ERG: multifocal Electroretinogram; CMT: Central macular thickness; IS/OS: Inner segment/outer segment; IOP: Intraocular pressure

A Spearman’s correlation analysis was used to explore the relationship between CDVA with mf-ERG amplitude & latency values, CMT, and the IS/OS junction integrity of the studied eyes, 6 months postoperatively.

**Fig. 3:**
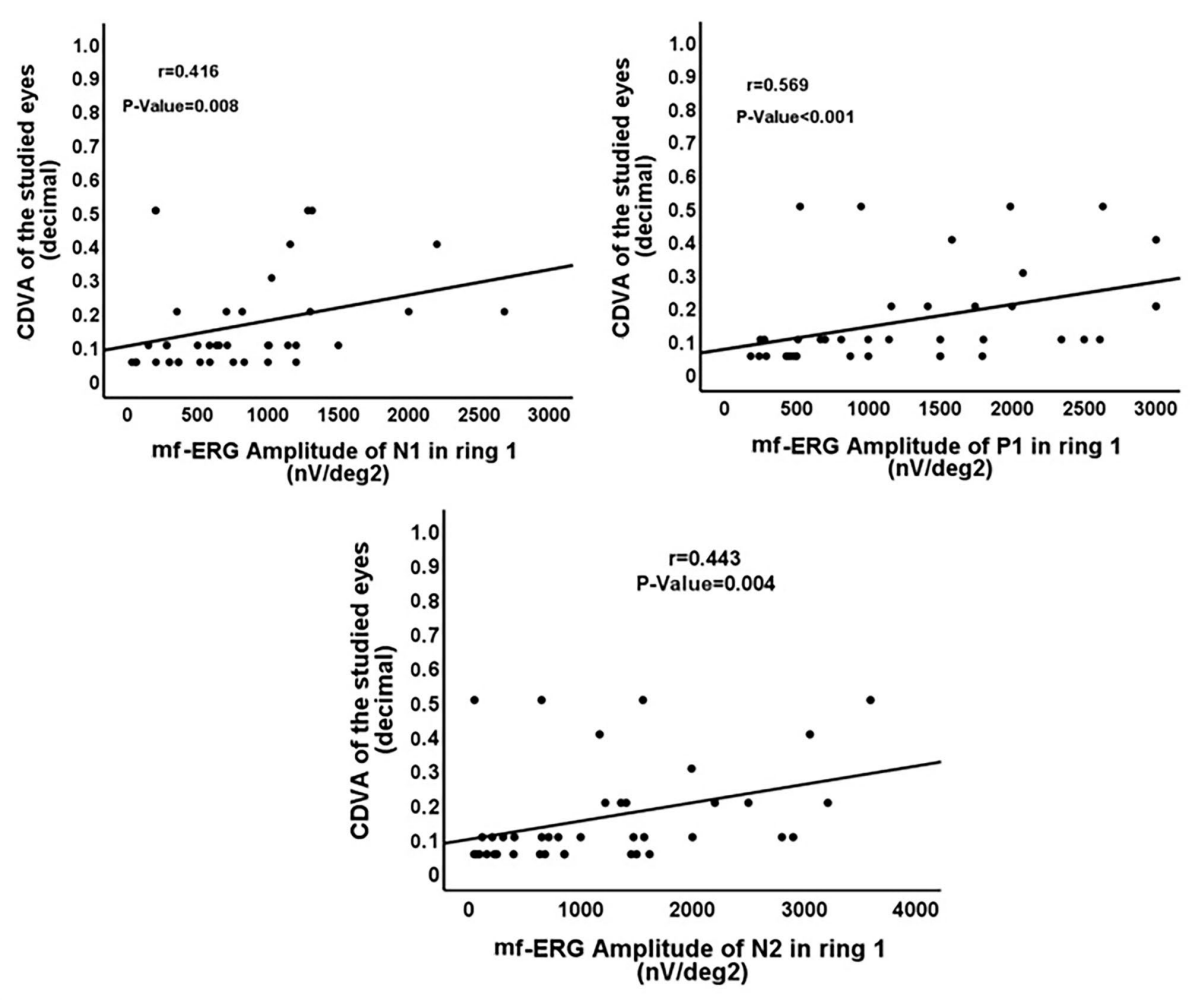
Correlation between 6-month postoperative CDVA with mf-ERG amplitudes of N1, P1, N2 in ring 1. (CDVA: Corrected distance visual acuity; mf-ERG: multifocal Electroretinogram)

**Fig. 4:**
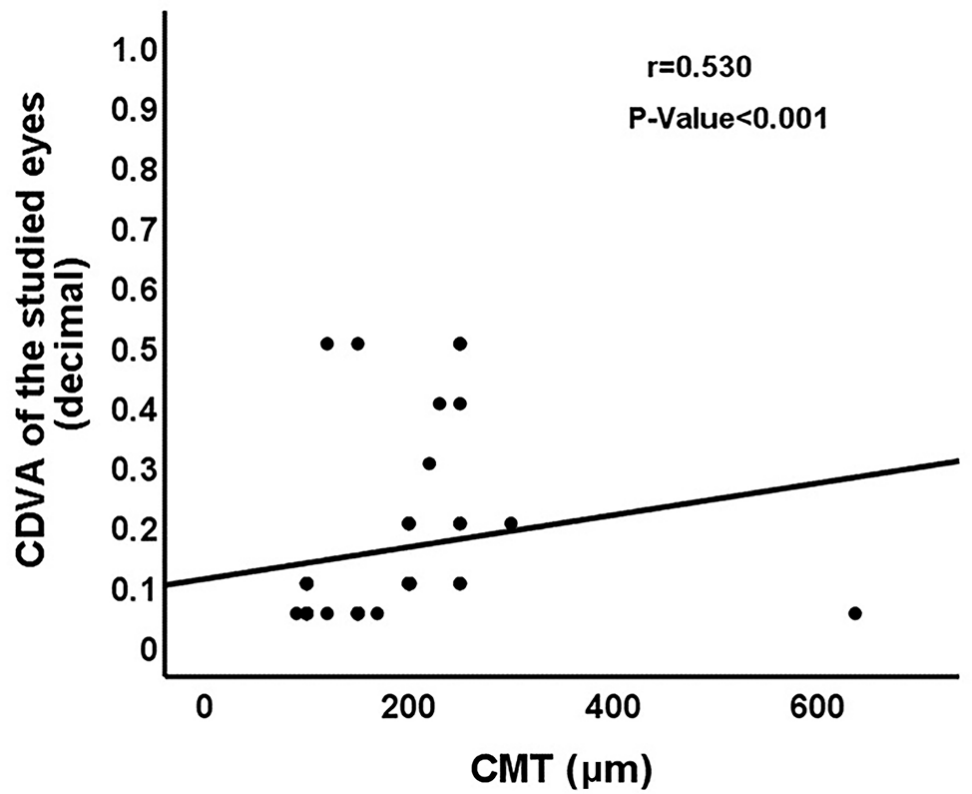
Correlation between 6-month postoperative CDVA and CMT. (CDVA: Corrected distance visual acuity; CMT: Central macular thickness)

**Fig. 5:**
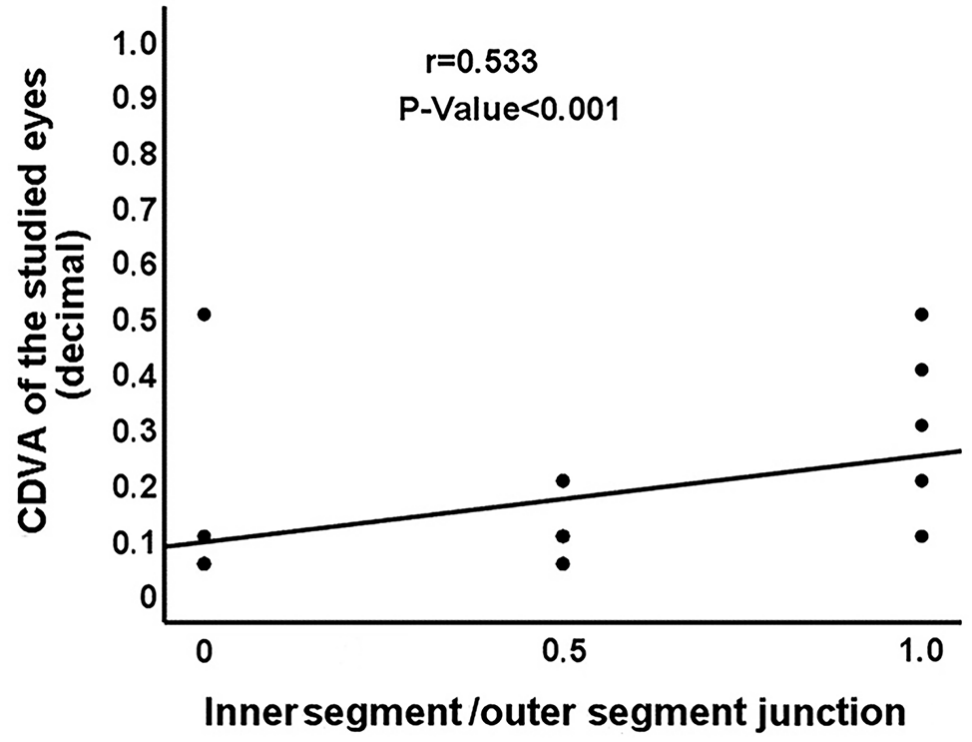
Correlation between 6-month postoperative CDVA and the IS/OS junction integrity. (CDVA: Corrected distance visual acuity; IS/OS: Inner segment/outer segment)

There was a statistically significant moderate positive correlation between CDVA of the studied eyes with mf-ERG amplitudes of N1, P1 and N2 in ring 1 (*P* = 0.008; *P* < 0.001; *P* = 0.004, Fig. 3), CMT (*P* < 0.001, Fig. 4), and the integrity of IS/OS junction (*P* < 0.001, Fig. 5). No significant correlation was found between the CDVA and mf-ERG latency (*P* > 0.05).

Similarly, a statistically significant moderate positive correlation was documented between the CMT of studied eyes with mf-ERG amplitudes of N1, P1 and N2 in ring 1 and the integrity of IS/OS junction (*P* < 0.001). The correlation between CMT with different mf-ERG latencies was non-significant (*P* > 0.05).

The correlations between the 6-month postoperative CDVA with mf-ERG amplitude & latency values, CMT, and the IS/OS junction integrity of the studied eyes are summarized in Table 2.

**Table 2:** Correlation between 6-month postoperative CDVA with mf-ERG amplitude & latency values, CMT, and the IS/OS junction integrity of the studied eyes

		CDVA	mf-ERG Amplitude of N1	mf-ERG Amplitude of P1	mf-ERG Amplitude of N2	mf-ERG Latency of N1	mf-ERG Latency of P2	mf-ERG Latency of N2	CMT
mf-ERG Amplitude of N1	r	**0.416**							
	*P*-Value	**0.008**							
mf-ERG Amplitude of P1	r	**0.569**	**0.912**						
	*P*-Value	**< 0.001**	**< 0.001**						
mf-ERG Amplitude of N2	r	**0.443**	**0.873**	**0.920**					
	*P*-Value	**0.004**	**< 0.001**	**< 0.001**					
mf-ERG Latency of N1	r	-0.060	0.115	0.217	0.297				
	*P*-Value	0.713	0.481	0.179	0.063				
mf-ERG Latency of P2	r	0.052	-0.256	− 0.171	-0.069	**0.329**			
	*P*-Value	0.749	0.111	0.293	0.670	**0.038**			
mf-ERG Latency of N2	r	0.149	-0.272	− 0.085	-0.047	0.274	**0.825**		
	*P*-Value	0.360	0.090	0.603	0.773	0.087	**< 0.001**		
CMT	r	**0.530**	**0.607**	**0.675**	**0.691**	0.144	0.111	0.115	
	*P*-Value	**< 0.001**	**< 0.001**	**< 0.001**	**< 0.001**	0.375	0.495	0.479	
IS/OS junction integrity	r	**0.533**	**0.762**	**0.748**	**0.813**	0.113	0.113	0.002	**0.710**
	*P*-Value	**< 0.001**	**< 0.001**	**< 0.001**	**< 0.001**	0.486	0.486	0.992	**< 0.001**

r (Spearman correlation)

Bold type signifies *P* ≤ 0.05

CDVA: Corrected distance visual acuity; mf-ERG: multifocal Electroretinogram; CMT: Central macular thickness; IS/OS: Inner segment/outer segment

Linear regression analysis revealed that 6-month postoperative CDVA was significantly associated with mf-ERG amplitudes and the IS/OS junction integrity while the association with CMT measures was non-significant (*P* = 0.293) as demonstrated in Table 3.

**Table 3:** Linear regression analysis for variables associated with changes in the visual acuity of participants

	CDVA			
	R^2^	ß	95% CI	*P*-Value
mf-ERG Amplitude of N1 in ring 1	**0.098**	**0.0001**	**0.0-0.0001**	**0.050**
mf-ERG Amplitude of P1 in ring 1	**0.163**	**0.000067**	**0.0-0.0001**	**0.010**
mf-ERG Amplitude of N2 in ring 1	**0.130**	**0.000054**	**0.0-0.0001**	**0.022**
CMT	0.029	0.00	0.000-0.001	0.293
IS/OS junction integrity	**0.164**	**0.016**	**0.004–0.027**	**0.010**

R^2^ (R square), ß: beta coefficient, 95% CI: 95% confidence interval (lower-upper)

Bold type signifies *P* ≤ 0.05

CDVA: Corrected distance visual acuity; mf-ERG: multifocal Electroretinogram; CMT: Central macular thickness; IS/OS: Inner segment/outer segment; CI: Confidence interval

There were statically significant differences (*P* value < 0.05) between diabetic and non-diabetic patients regarding the mean amplitudes of mf-ERG while there were no significant differences regarding the mean CDVA, mf-ERG latency, CMT, the IS/OS junction integrity.

## Discussion

Recent advances in the surgical techniques for treating RD have significantly improved the anatomical success rates of RD surgery exceeding 90% anatomical success rates [2, 10–12]. However, the visual outcomes after successful retinal reattachment remained relatively unsatisfactory with incomplete visual recovery and persistent postoperative visual dysfunction [10–12]. Functional visual recovery following RD surgery was reported to occur through regeneration of the photoreceptor outer segments and restoration of the contact between the retinal pigment epithelium and the neurosensory retina [13]. The poor visual recovery following retinal reattachment may be attributed to retinal ischemia, irreversible neuronal damage to the neurosensory retina, and microstructural retinal damage [10, 14].

In the current study, we evaluated the 6-month postoperative functional and anatomical outcomes of vitrectomy surgery for macula-off RRD. Postoperative CDVA had a significant positive correlation with mf-ERG amplitudes in ring 1, CMT, and the IS/OS junction integrity. However, no significant correlation was observed between CDVA and mf-ERG latencies in ring 1.

There is limited reference in the literature regarding the correlation between postoperative CDVA with mf-ERG and OCT findings following retinal reattachment surgery. Previous studies [6, 15–17] reported a significant improvement of ERG amplitudes following retinal reattachment. However, the recovery in ERG wave amplitudes was incomplete and the recorded amplitudes in the reattached retina remained lower than the amplitudes of the unaffected fellow eyes [6, 16]. A study by Wu et al. [15] reported that there was no significant change in the ERG latencies before and after RRD surgery. In the present study, we detected a moderate positive correlation between CDVA with mf-ERG amplitudes of N1, P1, and N2 in ring 1 but not with mf-ERG latencies. These findings are in accordance with observations of Wu et al. [15], Ricouard et al. [17] and Kominami et al. [18] who reported that despite the improvement of the amplitudes of focal macular ERG (FMERG), the delay in the implicit times of FMERG persisted over the 6-month follow-up after RRD surgery. Kominami et al. [18] proposed that this persistent increase in ERG latencies in spite of improved amplitudes may indicate an increase in the number but incomplete functional recovery of cone photoreceptors.

Regarding the OCT findings in our study, a positive correlation was detected between the postoperative CDVA and CMT (*r* = 0.530, *P* < 0.001) indicating that poor visual outcome and impaired retinal function are expected to occur with postoperative macular thinning associated with atrophy of the neurosensory retina. Hong et al. [19] reported that the central retinal thickness significantly decreased 6 months postoperatively compared with the fellow eyes, following vitrectomy for macula-off RRD. Rabina et al. [20] documented a transient reduction of CMT in patients who underwent RRD repair with silicon oil tamponade. After removal of silicone oil, an increase of CMT was observed that was associated with improvement of the postoperative visual acuity. A significant positive correlation was also detected between the postoperative CDVA and the IS/OS junction integrity (*r* = 0.533, *P* < 0.001). The correlation between the visual outcome following RD surgery and the IS/OS junction integrity was stressed by many studies [21–23]. Wakabayashi et al. [21] concluded that the postoperative CDVA was significantly correlated with the integrity of the photoreceptor IS/OS and ELM signals detected by Fourier-domain optical coherence tomography (FD-OCT) postoperatively (*r* = 0.805; *P* < 0.001). Additionally, they reported that the external limiting membrane integrity was a strong predictor of the restoration of IS/OS junction and subsequent visual recovery. Also, Saber et al. [22] documented a direct correlation between the postoperative macular microstructural changes detected by OCT and visual outcomes of retinal detachment repair. In addition to the significant correlation observed between the postoperative visual acuity and the IS/OS junction integrity (*r* = 0.786, *p* < 0.001), Abdussalam et al. [23] found that the IS/OS junction integrity and ELM disruption were significantly correlated with the duration of RRD thus emphasizing the importance of early surgery for RD. Not only the integrity of IS/OS junction but also the change in the outer nuclear layer thickness might be an important predictor of the visual outcome following RD repair as suggested by Gharbiya et al. [4].

Analyzing the correlation between the mf-ERG and OCT findings in our study, revealed a strong positive association between IS/OS junction integrity and the mf-ERG amplitudes of N1, P1, and N2 in ring 1. This association was stronger than the association between IS/OS junction integrity and CDVA suggesting that mf-ERG may be superior to CDVA in reflecting the extent of microstructural damage in the photoreceptor layer. Ricouard et al. [17] failed to detect any significant correlation between mf-ERG amplitudes and the inner segment ellipsoid band (ISe).

The mean amplitudes of N1, P1, and N2 were significantly lower in diabetic patients in the study compared with non-diabetic patients. The reduction of mf-ERG amplitudes and increased implicit times in diabetic patients compared to controls was similarly reported by other studies [24, 25]. Mohammed et al. [25] showed that the reduction in mf-ERG amplitude was proportionate to the duration of diabetes and was present even in the absence of diabetic retinopathy.

Limitations of the current study included the small sample size and the absence of baseline mf-ERG and OCT records for comparison with the postoperative results.

## Conclusions

The current study detected a significant correlation between multifocal electroretinogram and optical coherence tomography findings with visual acuity after vitrectomy surgery for macula-off rhegmatogenous retinal detachment. Furthermore, the combined use of postoperative CDVA, multifocal ERG, and OCT provides valuable information about functional visual recovery and retinal microstructural changes following RD surgery. The positive correlation between the IS/OS junction integrity and the mf-ERG amplitudes was stronger than the correlation between the IS/OS junction integrity and CDVA suggesting that mf-ERG may be superior to CDVA in reflecting the extent of microstructural damage in the photoreceptor layer.

## Data Availability

Data are available from the corresponding author on reasonable request.

## Abbreviations

mf-ERG: Multifocal electroretinogram
OCT: Optical coherence tomography
RD: Retinal detachment
RRD: Rhegmatogenous retinal detachment
CMT: Central macular thickness
CDVA: Corrected distance visual acuity
IS/OS: Inner segment/outer segment
IOP: Intraocular pressure
ELM: External limiting membrane
EZ: Ellipsoid zone
CME: Cystoid macular edema
ISCEV: International Society for Clinical Electrophysiology of Vision
FMERG: Focal macular electroretinogram
SPSS: Statistical package for social science
